# Examination of metabolic responses to phosphorus limitation via proteomic analyses in the marine diatom *Phaeodactylum tricornutum*

**DOI:** 10.1038/srep10373

**Published:** 2015-05-28

**Authors:** Tian-Ya Feng, Zhi-Kai Yang, Jian-Wei Zheng, Ying Xie, Da-Wei Li, Shanmugaraj Bala Murugan, Wei-Dong Yang, Jie-Sheng Liu, Hong-Ye Li

**Affiliations:** 1 Key Laboratory of Eutrophication and Red Tide Prevention of Guangdong Higher Education Institutes, College of Life Science, Jinan University, Guangzhou 510632, China

## Abstract

Phosphorus (P) is an essential macronutrient for the survival of marine phytoplankton. In the present study, phytoplankton response to phosphorus limitation was studied by proteomic profiling in diatom *Phaeodactylum tricornutum* in both cellular and molecular levels. A total of 42 non-redundant proteins were identified, among which 8 proteins were found to be upregulated and 34 proteins were downregulated. The results also showed that the proteins associated with inorganic phosphate uptake were downregulated, whereas the proteins involved in organic phosphorus uptake such as alkaline phosphatase were upregulated. The proteins involved in metabolic responses such as protein degradation, lipid accumulation and photorespiration were upregulated whereas energy metabolism, photosynthesis, amino acid and nucleic acid metabolism tend to be downregulated. Overall our results showed the changes in protein levels of *P. tricornutum* during phosphorus stress. This study preludes for understanding the role of phosphorous in marine biogeochemical cycles and phytoplankton response to phosphorous scarcity in ocean. It also provides insight into the succession of phytoplankton community, providing scientific basis for elucidating the mechanism of algal blooms.

Phosphorus (P) is indispensable for the structure and function of all the living organisms. It is also an essential nutrient for the survival of marine phytoplankton. In the living system, phosphorous is mainly involved in biological energy transfer mechanisms and cell growth. Phosphate ester constitutes the skeleton for the formation of DNA and RNA. It is the major component of cell membranes in the form of phosphorus-containing proteins and phospholipid; also energy transfer in the form ATP^1^. Utilization of phosphorus in seawater affects the nutritional status, cell volume, photosynthetic efficiency and other metabolic activities of phytoplankton^2^,^3^ , thereby affects the composition and quantity of phytoplankton community^1^,^4^. Therefore, the bioavailability of phosphorus is closely related to marine primary production, carbon cycle and nitrogen fixation^5^,^6^,^7^. In recent years, several studies reported that the phosphorus is a limiting nutrient rather than nitrogen in the major marine ecosystems in the long-term geological period^8^,^9^. The primary production in marine water are in a state of phosphorus limitation, such as the Northwest Atlantic^10^, North Pacific^11^, Eastern Mediterranean^12^,^13^,^14^, and Chinese coast^15^,^16^,^17^, etc. Even in nitrogen limited ecosystem, some species of phytoplankton are also exposed to phosphorus limitation^4^,^18^.

Phytoplankton is one of the most important producers in marine food chain. Nutrient limitation has been found to have different effects on cell growth rate, size, pigment composition, density and lipid content in microalgae^19^,^20^,^21^,^22^,^23^. As it is a photosynthetic organism, marine diatoms provide a large amount of organic food to marine organisms^24^. The pennate diatom *Phaeodactylum tricornutum* has been a model organism for research in diatoms. Considering inadequate data available on P uptake mechanisms and its response to P starvation, this study was designed to study the metabolic network shifts of diatom under P limitation (–P) and to reveal any new adaptive the alternative metabolic pathways adapted by diatoms during –P depletion. Recently, transcriptional changes of *P. tricornutum* under -P stress has been elucidated and revealed a number of genes involved in response to –P stress^25^. In this study, proteomic analysis was used to evaluate the changes at protein level in order to further understand the molecular mechanism behind –P stress in *P. tricornutum*.

## Results and Discussion

### Differentially expressed proteins under -P stress

**Figure 1:**
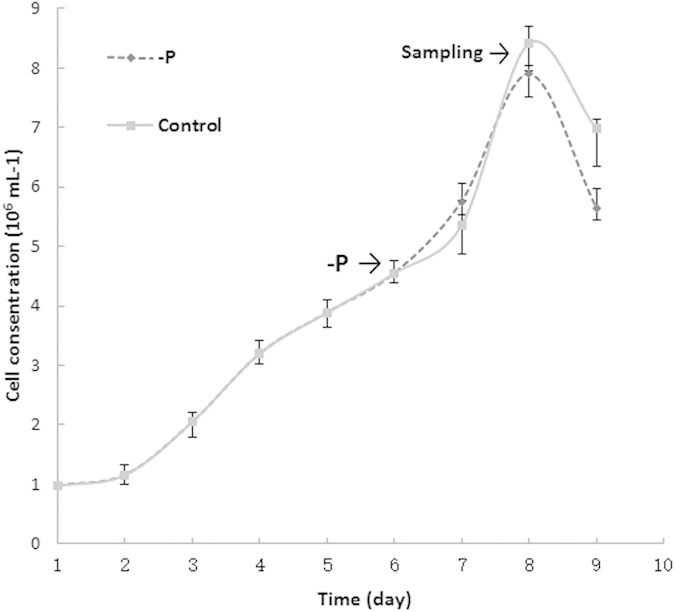
Time points of –P treatment and protein sampling.Six days after subculture *P. tricornutum* cells were treated with –P. Total protein was extracted from cultures at 48 h after –P treatment.

**Figure 2:**
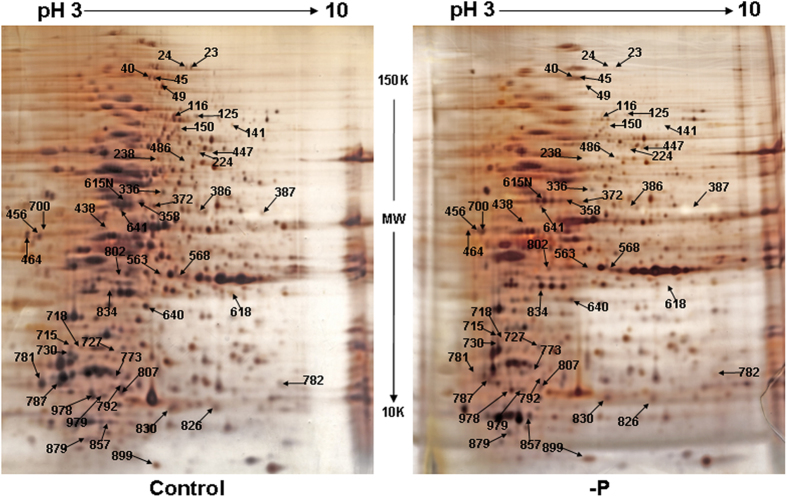
Representative 2-DE gels of diatom proteins of P-replete (control) and P-deprived (-P) cultures. Left: control; right: -P. Molecular weight and pH are indicated at the side and top of gels. Spot numbers are corresponding to that in Table 1.

*P. tricornutum* cells were treated with –P after 6 days of subculture, and the cells were harvested for proteomic analysis after 48 h of treatment (Fig. 1). Protein-level changes in *P. tricornutum* in response to –P depletion and control cultures were analyzed by 2-D electrophoresis (2-DE). Almost 1,000 spots were automatically matched between the gels. A total of 58 differentially expressed spots with a volume ratio of >2.0 (p < 0.05) were successfully identified (Fig. 2) including 42 non-redundant proteins (Table 1). Among them, 8 were upregulated and 34 were downregulated. The results from 2-DE experiments were further validated by qPCR performed on a set of 8 different proteins. As shown in Table 2, 8 out of the 10 randomly selected proteins showed consistent up-/down-regulation between qPCR and 2-DE results. However, 2 proteins showed an inconsistency between qPCR and 2-DE results including PHATRDRAFT_42406 and 12583, suggesting that the relationship between mRNA as evaluated by qPCR and protein as evaluated by 2-DE is not always straight-forward.

**Figure 3:**
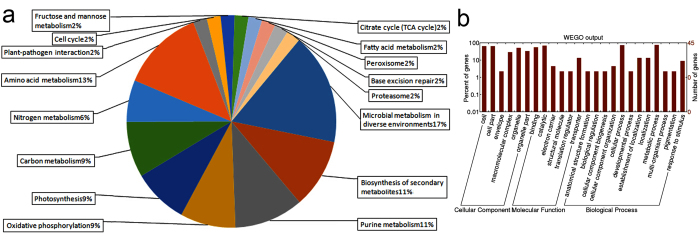
Functional categories of differentially expressed proteins involved in various biological processes.Proteins identified were mapped to KEGG pathways by using KEGG database and homology mapping (**a**) and WEGO for plotting GO annotation results (**b**).

The identified proteins were mapped onto KEGG (Kyoto Encyclopedia of Genes and Genomes) pathway database and GO (Gene Ontology) annotations to reveal the metabolic responses of *P. tricornutum* in response to –P depletion. The mapping results (Fig.3) showed that almost 41 proteins were involved in cellular and physiological mechanisms including primary metabolic pathways associated with amino acid, nucleic acid and lipid metabolism; also other pathways such as photosynthesis, synthesis of secondary metabolites, etc. Among the identified proteins, no annotation was found for the predicted protein PHATRDRAFT_49815. Detailed analysis of the differentially expressed proteins and altered metabolic pathways were described below.

### Proteins associated with absorption and utilization of phosphorus

Phytoplankton possesses the ability to limit their phosphorus demand and maintain their growth even during –P scarcity^26^; however, its biochemical mechanism behind this process is still unknown. The earlier reports showed that the scarcity of inorganic phosphorus may lead to series of changes in the cell membrane^25^ which is in accordance with our results. The results of our study showed that some membrane proteins involved in the transport of inorganic phosphorus were decreased. Permease (PHATRDRAFT_49038) and an unknown protein (PHATRDRAFT_33928) which was predicted to have ATPase activity and transmembrane transport, showed significant downregulation. Permease is a class of multipass transmembrane proteins which helps in assisting the molecules to transport in and out of the cell^27^. Due to the downregulation of transport proteins, the phosphorus uptake by the cells was significantly reduced. As a result of lack of inorganic phosphorus, the cell might overcome the -P stress by induction of alkaline phosphatase activity thereby cleaving various phosphate monoesters on the cell surface, which aids the cells to use organic phosphate source when inorganic phosphate is no longer available^28^. Correspondingly, a significant upregulation of alkaline phosphatase D (phoD, PHATRDRAFT_45757) was observed in our study, which provides a clue that algal cells might try to use the organic phosphorus to reduce the stress caused by inorganic P limitation. Therefore, the presence of alkaline phosphatase has become a common biochemical indicator of phytoplankton species growing under -P depletion^29^. *P. tricornutum* can able to grow at least for two generations under –P lacking environment, however, P content in the progeny was found to be significantly decreased (46%) after 48 h of cells growing in –P depleting conditions^25^. Our results proved that the algal cells in –P starvation might use the intracellular P to supply the progenies in further generations, resulting in a decreased P content in the progenies. Earlier reports showed that phytoplankton can replace membrane phospholipids with non-phosphorus lipids to reduce their cellular phosphorus requirements^30^. Meanwhile phospholipid turnover could provide sufficient P source for cell growth^31^.

### Upregulation of stress-shock proteins

A series of changes of stress-responsive proteins were observed during oxidative stress caused by –P in cells. Superoxide dismutase (SOD) catalyzes the dismutation of superoxide (O^2−^) into hydrogen peroxide and oxygen. It plays a major role in the antioxidant defense mechanism when the cells are exposed to molecular oxygen^32^. Accordingly, superoxide dismutase (SOD2-PHATRDRAFT_12583) was upregulated under –P depletion. However, there is an inconsistency between qPCR (downregulation of PHATRDRAFT_12583, Table 2) and 2-DE results, possibly due to the translational regulation and/or posttranslational modification of the protein. Some proteins showed downregulation which may be due to cell damage or lack of function in algal cells upon –P depletion such as proteins involved in energy metabolism or photosynthesis. Intracellular membranes were found to be disrupted and poorly organized in *P. tricornutum* cells under –P stress^25^. Partially degraded intracellular membranes may cause degradation of membrane proteins. Generally, there are two ways of protein degradation, *i.e.,* ubiquitin-proteasome pathway and lysosomal pathway, which can yield short peptides for further use in synthesizing new proteins^33^. We have observed an upregulation of phosphomannomutase (PMM, PHATRDRAFT_28882), whose catalytic product mannose 6-phosphate^34^ is essential in targeting acid hydrolases to the lysosome or lytic vacuole for their proper functions^35^. Hence, we hypothesize that the algal cells can prevent further damage by enhancing the degradation of unwanted proteins. Also, we noticed that LRR receptor-like serine/threonine protein kinase FLS2 (PHATRDRAFT_44441) corresponding to signal transduction was upregulated. The receptor-like kinase (RLK) gene family in plants is thought to have accelerated evolution of domains presumably involved in signal reception, especially the leucine-rich repeat (LRR). Accordingly, the RLK plays an essential role in pathogen recognition and the subsequent activation of series of plant defense mechanisms and developmental control^36^. However, sorting nexin (PHATRDRAFT_3137) functioning in cell communication was found to be downregulated. Sorting nexins are a large group of proteins localized in the cytoplasm, which have potential in membrane association through either by phospholipid-binding domain or by protein-protein interactions with membrane-associated protein complexes and few members of this protein family facilitates protein sorting^37^.

### Downregulation of proteins involved in photosynthesis

Normally proteins associated with photosynthesis were mainly affected proteins during nutrient stress. It has been reported that -P stress could destroy the PSII process and reduce the chlorophyll fluorescence parameter Fv/Fm (the ratio of the variable/maximum fluorescence)^38^ which is consistent with our findings. In our study, the expression of some proteins which play an important role in photosynthesis were reduced, including photosystem I subunit VII (psaC, PSAC_PHATC), photosystem II cytochrome c550 (psbV, CY550_PHATC), fucoxanthin chlorophyll a/c protein (PHATRDRAFT_17766, PHATRDRAFT_54065, PHATRDRAFT_22680, PHATRDRAFT_25168, PHATRDRAFT_50705, PHATRDRAFT_18049), F-type H+-transporting ATPase beta subunit (ATPF1B, ATPB_PHATC), and gamma subunit (ATPF1G, PHATRDRAFT_20657). During -P stress, transcription levels of genes related to photosynthesis was found to be increased^25^. Such differences implicated that, during –P stress, photosystem of *P. tricornutum* was damaged at a certain limit, in order to maintain normal physiological function and also cells promoted transcription of photosynthesis-related proteins to recover from the damage. However, due to the time difference between transcription and translation, the increased transcripts have not translated into proteins at the sample collecting time point, thus there would be increased transcript abundance with decreased corresponding translated proteins at the certain sampling time. However, surprisingly, the parameter Fv/Fm increased slightly in the cells during the stationary phase after 48 h of -P treatment^25^. This indicates that photosystem was damaged only to a minor extent during early stages of –P depletion which is not severe enough to affect the light harvesting efficiency. However, with further damage on the photosystem, photosynthetic efficiency would inevitably decline. We observed that the expression of ribulose-1, 5-bisphosphate carboxylase/oxygenase (PhtrCp060) was increased; implicating that carbon assimilation could be elevated^39^. Reports proved that carbon (C) content found to be slightly increased by 3% in *P. tricornutum* cells after 48 h of –P treatment and *P. tricornutum* could grow at least for further two generations under –P^25^. Moreover, carbonic anhydrase (PHATRDRAFT_42406) which plays an important role in CO_2_ concentration mechanism (CCM), showed upregulation, suggesting that the reversible inter-conversion of carbon dioxide and water to bicarbonate and protons (or vice versa) between cells and the outside was promoted^40^. It implicates that the carbon fixation still continued after -48 h of –P starvation even though photosynthetic efficiency started to decline.

The results showed that the growth of algal cells could not be affected much in a shorter period after –P depletion. However, after certain limit, the cells was metabolically altered and severely damaged, which is in accordance with the previous findings which proved that intracellular membranes were disrupted and poorly organized under –P stress^25^. This is in consistent with the growth curve where little difference was observed in the early stage of -P compared to the control sample, but in the later stages, the cells growing under –P depletion decay faster than the control culture. It was further supported by non-significant difference in Fv/Fm values between –P and control algal cells^25^.

### Upregulation of proteins associated with lipid accumulation

In most microalgal species, lipid accumulation usually occurs in order to adapt to the environmental stress and cultivation conditions. P limitation has been already reported to induce lipid accumulation in a few microalgae species^19^,^20^. *P. tricornutum* was previously found to show 60% increase in neutral lipid content after 48 h of –P stress and intracellular membranes were disrupted and disorganized^25^. Accordingly, in our study, annexin (PHATR_44109) showed downregulation. Annexins play an important role in providing a membrane scaffold^41^, and also involved in trafficking and organization of vesicles^42^. In our study, 11 genes encoding putative phospholipases that have roles in phospholipid degradation showed increased transcription in *P. tricornutum* under -P^25^, therefore, the downregulation of annexin implicated that algal cells began to acclimate to the disruption of their membrane integrity and cellular structure. The degradation of membrane phospholipids not only provided raw material for lipid accumulation, but also supplied P for cell growth. Also, we observed the downregulation of enoyl-CoA hydratase (PHATRDRAFT_55192), which is responsible for hydrating the double bond between the second and third carbons on acyl-CoA, also known as crotonase important in catalyzing fatty acids to produce acetyl-CoA and energy^43^. The downregulation of enoyl-CoA hydratase could reduce fatty acid catabolism but promoted anabolic pathways thus resulting in lipid accumulation. Moreover, we found an upregulation of putative PAP fibrillin (PHATRDRAFT_45813), a kind of plastid-lipid-associated protein^44^. Proteomic profiling of oil bodies was performed in green microalga *Chlamydomonas reinhardtii*^45^ and reported that the micro-fiber protein was associated with lipid accumulation under –N stress, primarily for maintaining the stability of oil bodies in the algal cells. Oil body is an organelle for storing neutral lipids^46^; thus, the upregulation of PAP fibrillin could help in maintaining the oil bodies in *P. tricornutum* under –P stress. In fact, the number and sizes of oil bodies were found to be increased in *P. tricornutum* under –P stress^25^.

### Proteins associated with nucleic acid metabolism

Nucleic acids contain approximately 9% P dry mass^47^. Therefore, under –P starvation, cells would adopt the most economical way to recycle the existing nucleic acids. We observed that the proteins related to nucleotide biosynthesis were downregulated, such as carbamate kinase (PHATRDRAFT_24238), adenylosuccinate synthetase (PHATRDRAFT_26256) and putative phosphoribosyl aminoimidazole carboxylase (PHATRDRAFT_56626). Carbamate kinase (CKase) is a member of transferases, transferring phosphorus-containing groups with a carboxyl group as an acceptor, which is involved in purine metabolism and other metabolic pathways such as arginine and proline metabolism, nitrogen metabolism and glutamate metabolism^48^,^49^. Adenylosuccinate synthase and phosphoribosylaminoimidazole carboxylase are important in purine biosynthesis but have different functions^50^. Adenylosuccinate synthase catalyses the guanosine triphosphate (GTP)-dependent conversion of inosine monophosphate (IMP) and aspartic acid to guanosine diphosphate (GDP), phosphate and N(6)-(1,2-dicarboxyethyl)-AMP^51^. Phosphoribosylaminoimidazole carboxylase converts 5'-phosphoribosyl-5-aminoimidazole into 5'-phosphoribosyl-4-carboxy-5-aminoimidazole^52^. Similarly, enzymes involved in nucleotide catabolism were reduced, including putative urease (PHATRDRAFT_29702) which regulates purine catabolism^53^.

### Downregulation of proteins associated with amino acid metabolism

Proteins associated with amino acid metabolism was found to be downregulated after 48 h of -P starvation, including carbamate kinase (PHATRDRAFT_24238) and urease (PHATRDRAFT_29702) involved in arginine and proline metabolism, glutamate synthase (NADPH/NADH) small chain (PHATRDRAFT_20342) and adenylosuccinate synthase (PHATRDRAFT_26256) involved in alanine, aspartate and glutamate metabolism, and enoyl-CoA hydratase (PHATRDRAFT_55192) involved in tryptophan metabolism. Among these, downregulation of glutamate synthase would cause a reduction of glutamate which plays an important role in transamination of amino acids in amino acid metabolism^54^. The downregulation of amino acid metabolism suggested that the cells started to reduce their cellular activities after -48 h of –P depletion. Moreover, the downregulation of amino acid metabolism would result in the reduced amount of ammonia in the cell, which is consequent with the downregulation of urease, indicating that the urea cycle tends to stagnate as well.

### Proteins associated with energy metabolism

Earlier reports showed that phytoplankton have the ability to lower their physiological demand of phosphorus by about 50% during P deficiency^30^, thus algae could grow at least two generations^25^. This indicates that *P. tricornutum* can managed their cellular metabolism during the early stages –P depletion. Hence, we assumed that the cellular activities during early stage might consume large amount of P in the cell. P content in the offspring showed a significant decrease (46%) after the growth of further two generations in the –P depleted medium^25^, indicating that the cells consumed the intracellular P such as membrane phospholipids^31^ . However, after 48 h of –P stress, algal cells accumulate lipids to a maximum level, while P in cells was consumed to its lowest point^25^. As a result, the cellular metabolic activities were reduced. Thus, most of proteins involved energy generation process began to downregulate such as ATP synthase beta subunit (ATPB_PHATC), ATP synthase gamma subunit (PHATRDRAFT_20657), putative NADPH:quinone reductase and related Zn-dependent oxidoreductase (PHATRDRAFT_18893) involved in photosynthetic phosphorylation pathway, and succinyl CoA synthetase (PHATRDRAFT_42015) involved in the pathway of substrate level phosphorylation. Succinyl CoA synthetase (SCS, also called succinate-CoA ligase or succinate thiokinase) is an enzyme catalyzing the reversible conversion of succinyl-CoA and succinate coupling of the formation of a nucleoside triphosphate molecule (either ATP or GTP) from a nucleoside diphosphate molecule (either ADP or GDP)^55^. It is located in the mitochondrial matrix as one of the catalysts that plays an essential role in citric acid cycle^56^. Moreover, ubiquinone oxidoreductase (PHATR_43944) and ATP synthase mitochondrial subunit (PHATRDRAFT_54086) involved in the oxidative phosphorylation pathway was downregulated except a vacuolar ATP synthase subunit b (PHATRDRAFT_24978). These results showed that most of the proteins were downregulated, hence we deduce that most of the metabolic activities were ceased in the cells after 48 h of –P depletion.

### Proteins associated with translation process, folding and modification

The results showed that the two proteins involved in translation process were downregulated; the putative elongation factor 3 (EF-3) (PHATRDRAFT_27838), which facilitates EF-1-alpha-dependent binding with aminoacyl-tRNA to the ribosome, thus resulting in the transduction of mechanical energy from nucleoside triphosphate energy for translocation during translation^57^ and chloroplast elongation factor Ts, (EFTS_PHATC), which associates with the EF-Tu-GDP complex and remains bound to the aminoacyl-tRNA, thus facilitating the conversion of GDP to GTP^58^. This showed that the translation levels of proteins decreased after 48 h of –P starvation. Meanwhile, a putative FKBP-type peptidyl-prolyl cis-trans isomerase (PHATRDRAFT_12411) associated with protein folding, was downregulated. Moreover, most of the proteins involved in protein modification exhibited a significant downregulation, for instance, n-acetylglucosaminyl transferase-like protein (PHATRDRAFT_47316) associated with glycosylation, aspartyl asparaginyl beta-hydroxylase (PHATRDRAFT_44505) involved in the peptidyl-amino acid modification pathway, and a member of the AAA+ (ATPases associated with a wide variety of cellular Activities) superfamily predicted to have function in posttranslational modification, protein turnover and chaperones. AAA+ proteins play a major role in transducing chemical energy that are produced by conformational changes during ATP hydrolysis^59^.

### Proteins associated with nucleic acid metabolism

ESCO2 (establishment of cohesion 1 homolog 2, PHATRDRAFT_43362) involved in regulation of DNA replication was found to be downregulated. During the S phase of mitosis, ESCO2 was involved in the establishment of sister chromatid cohesion with acetyltransferase activity^60^. Hence, we deduced that DNA replication was greatly affected in the cells leading to abnormal cell division, which corresponds with the observation that *P. tricornutum* growing under -P depletion decayed faster than the control one^25^. Therefore, proteins associated with base excision repair would be downregulated, which is consistent with the observation of downregulation of high mobility group protein B1 (HMGB1, PHATRDRAFT_24886). HMGB1 that coordinates the DNA transcription by interacting with nucleosomes, histones and transcription factors^61^,^62^.

## Conclusion

**Figure 4:**
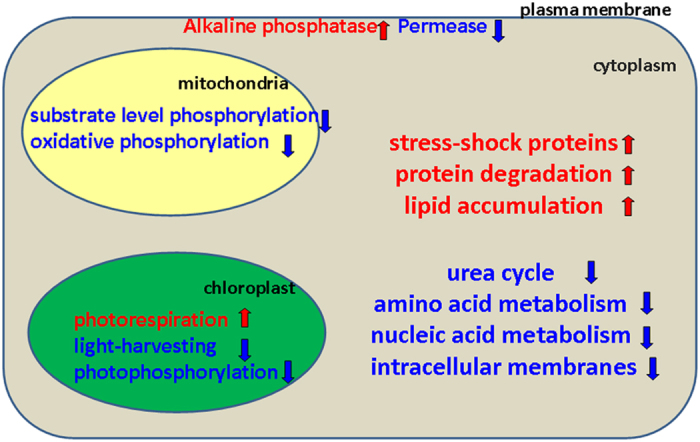
Metabolic responses of *P. tricornutum* under –P stress.The up and down arrows indicate the upregulated (red) and downregulated (blue) metabolic pathways, respectively.

In summary, both cellular and molecular response of diatom *P. tricornutum* under P limitation was studied. The results showed the blueprint of upregulated and downregulated proteins in *P. tricornutum* under –P stress which revealed the diverse biochemical strategies that are likely to be involved to overcome P limitation (Fig. 4). In addition, we further elucidated the molecular mechanisms of P uptake under –P stress, thereby improving our knowledge on the role of P in marine biogeochemical cycles and phytoplankton response to P limitation in marine environment.

## Methods

### Algal culture conditions

*Phaeodactylum tricornutum* used in the present study was obtained from the Freshwater Algae Culture Collection, Wuhan, China (No. FACHB-863). The diatom was maintained in filter-sterilized f/2-Si medium (f/2 medium without Na_2_SiO_3_•9H_2_O) as batch cultures in flasks. The diatom was subcultured every 7 days in an artificial climate incubator at a constant irradiance (200 μmol photons m^–2^·s^–1^) and temperature (21 ± 0.5 °C) with 12 h/12 h (1ight/dark) photoperiod cycle. Cell concentration (cells mL^−1^) was measured in triplicate with a Brightline Hemocytometer under an Olympus microscope (Olympus, Japan) at regular times each day. After the culture reached stationary phase (6 days), 1.5 L culture was divided into two halves, one of which was used to study –P stress, and the other half was used as control with P replete culture. The cultures were centrifuged at 2500 g for 10 min at 4 °C and the pellet was washed twice with medium lacking -P (without the NaH_2_PO_4_·2H_2_O). After centrifugation at 2500 g for 15 min, the pellet was resuspended in 1.5 L of medium that lacks -P. Half of the 1.5 L culture was supplemented with P replete medium. All the cultures were transferred into flasks containing 125 mL culture each used as 6 replicates of –P and P-replete cultures, respectively.

### Protein extraction and 2-D electrophoresis

The diatom samples from the treated and control samples (a total of 6 simultaneously grown cultures used per sample with three biological replicates) were pelleted by centrifugation at 2500 g for 10 min at 4 °C. Pelleted cells were ground into a fine powder with liquid N_2_. The powder was transferred into a 1.5 mL tube, mixed with 500 μL of lysis buffer and incubated at 4 °C for 30 min. After removing the cellular debris by centrifugation at 15,000 × g for 30 min at 4 °C, pre-chilled acetone (5 times volume) was added and incubated at -20 °C for 1h to precipitate the proteins. Crude protein was obtained by centrifugation as above. The protein precipitate was rinsed three times with pre-chilled acetone and similarly recovered by centrifugation as above. Finally, the pelleted protein was solubilized completely in 200 μL rehydration buffer. The insoluble residues were removed by centrifugation as above. Protein concentration was measured by Bradford assay (BioRad, USA). 2-D electrophoresis was performed according to Yajima *et al.*^63^ by using an Ettan IPGphor III Isoelectric Focusing System (GE, USA). An aliquot of 200 μg protein was taken from each replicate for passive rehydration on a Ready Strip IPG strips pH3-10 NL (GE, USA).

### 2-DE protein identification

Silver-stained gels were scanned with an image scanner and analyzed by using Image Master 2D Platinum 6.0 software (GE, USA). Significant protein spots (Student’s *t* test, p < 0.05) with an abundance ratio of two or more were chosen as differentially expressed proteins. The corresponding spots were excised from the gels manually; in-gel trypsin digestion and MS analysis was performed following the method described by Zhao *et al.*^64^. The identified proteins were matched to specific functions or processes by querying Gene Ontology (GO) online ( http://www.geneontology.org/). The change in the metabolic pathways due to –P starvation was determined by using GO term clustering in KEGG pathway database ( http://www.genome.jp/kegg/) and BGI WEGO (Web Gene Ontology Annotation Plotting, http://wego.genomics.org.cn/cgi-bin/wego/index.pl).

### Verification of expression of proteins identified in 2-DE by qPCR

Transcription of identified proteins was measured by quantitative real-time PCR (qPCR, conducted in Boxin Ltd., Guangzhou, China). Total diatom RNA was extracted and reverse-transcribed with random hexamer primers using an Omniscript reverse transcription kit (QIAgen, Germany). Reactions were performed in 96-well plates with 20 μL mixture per well, using a SYBR Green Kit and a 7300 Sequence Detection System (Applied Biosystems, USA). Primers used were listed in Table 2 which were specific to the corresponding genes. The predicted Actin like protein (ACT1, Phatrdraft_51157) of *P. tricornutum* was used as housekeeping marker control. Primers for ACT1 were (Forward: 5`-TTCCAGACCATTATGAAGTGCG-3`) and (Reverse: 5`-TGACCCTCCAATCCAAACAGA-3`) which could generate a 198-bp product. The Ct (threshold cycle) for each well was measured, and relative mRNA levels were quantified by normalization to β-actin.

## Additional Information

**How to cite this article**: Feng, T.-Y. *et al.* Examination of metabolic responses to phosphorus limitation via proteomic analyses in the marine diatom *Phaeodactylum tricornutum*. *Sci. Rep.* **5**, 10373; doi: 10.1038/srep10373 (2015).

## Figures and Tables

**Table 1:** List of significantly regulated proteins in P-deprived diatom cells.

Locus tag	Spot ID	Symbol	Annotation	Protein mass	pI	Protein Score	Coverage	FC
**Upregulated proteins**								
PHATRDRAFT_12583	834	SOD	precursor of mutase superoxide dismutase [Fe/Mn]	24336.8	4.92	61.5	49.54	++++
PHATRDRAFT_24978	447	ATPeVB	putative proton-transporting two-sector ATPase complex	55944.61	6.15	344	62.45	+
PHATRDRAFT_28882	802	PMM	phosphomannose mutase	28501.23	4.92	88.6	24.50	++++
PHATRDRAFT_42406	568	CA	intracellular beta-type carbonic anhydrase	31344.05	6.87	121	11.35	+
PHATRDRAFT_44441	879	FLS2	predicted leucine-rich repeat receptor-like protein kinase	501903.4	4.61	59.4	5.04	+
PHATRDRAFT_45757	486	phoD	putative metallophosphatase	58978	5.93			++++
PHATRDRAFT_45813	700	PAP	putative PAP_fibrillin	33585.91	4.28	66.0	17.0	++++
PhtrCp060	979	rbcS	ribulose-1,5-bisphosphate carboxylase/oxygenase small subunit	16240.83	5.23	111	34.53	+
**Downregulated proteins**								
ATPB_PHATC	438	ATPF1B	ATP synthase beta subunit, chloroplastic	51588.79	4.45	289	27.16	-
ATPG_PHATR	387	ATPF1G	chloroplast ATPase gamma subunit precursor	40188.1	9.47	123	22.49	----
CY550_PHATC	826	psbV	photosystem II cytochrome c550	17825.28	8.21	165	29.45	--
EFTS_PHATC	830	EFTS	elongation factor Ts, chloroplastic	23143.04	5.49	313	70.30	--
PHATRDRAFT_17766	730	Lhcr4	fucoxanthin chlorophyll a/c protein	23280.48	4.52	78.9	17.21	--
PHATRDRAFT_12411	978	PPlase	putative FKBP-type peptidyl-prolyl cis-trans isomerase	13455.92	4.46	73.2	47.20	--
PHATRDRAFT_18049	781	FCPA	Fucoxanthin-chlorophyll a-c binding protein A, chloroplastic	21315.84	4.79	140	10.71	----
PHATRDRAFT_18893	336	NQO	putative NADPH:quinone reductase and related Zn-dependent oxidoreductases	38668.89	6.26	245	34.16	--
PHATRDRAFT_20342	150	gltD	glutamate synthase	65407.33	6.33	63.6	16.90	----
PHATRDRAFT_20657	386	ATPG	precursor of ATPase gamma subunit	40124.07	9.29	81.9	17.34	--
PHATRDRAFT_22680	49	Lhcf13	fucoxanthin chlorophyll a/c protein	21573.17	4.46	69.6	15.74	----
PHATRDRAFT_24238	372	arc	carbamate kinase	35956.75	5.29	71.9	26.49	----
PHATRDRAFT_24886	618	HMG-2	putative high mobility group protein B2	23364.88	9.07	94.1	25.00	----
PHATRDRAFT_25168	782	Lhcf4	fucoxanthin-chlorophyll a/c light-harvesting protein	13758.11	4.59	69.4	16.80	--
PHATRDRAFT_26256	224	ADSS	Adenylosuccinate synthetase	57563.92	6.65	59.5	12.17	--
PHATRDRAFT_27838	23	EF-3	putative elongation factor 3 (EF-3)	115686.6	6.02	62.1	14.62	----
PHATRDRAFT_29702	45	URE	putative urease	94486.65	6.02	80.8	3.99	-
PHATRDRAFT_3137	141	SNX	predicted phosphoinositide binding Phox Homology domain of Sorting Nexins 1 and 2	44660.49	6.97	66.6	34.44	---
PHATRDRAFT_33928	807	ATPase	predicted ATPase activity and related to transmembrane transport	22416.78	5.39	100	33.33	--
PHATRDRAFT_42015	358	sucD	succinate-CoA ligase	32783.49	5.03	61.8	13.27	----
PHATRDRAFT_42434	116	ATPase	putative ATPases of the AAA+ class	52465.2	6.44	84.3	12.19	---
PHATRDRAFT_43362	727	ESCO2	predicted ESCO2 (establishment of cohesion 1 homolog 2)	44713.3	10.21	62.5	21.35	--
PHATRDRAFT_43944	238	NDUFV1	putative NADH dehydrogenase (ubiquinone) flavoprotein 1	54760.86	6.07	61	19.68	----
PHATRDRAFT_44109	641	ANX	annexin	37422.81	4.92	184	26.97	-
PHATRDRAFT_44505	715	ASPH	predicted Aspartyl/Asparaginyl beta-hydroxylase	59999.7	6.28	51	11.13	--
PHATRDRAFT_47316	857	GT	predicted Glycosyl transferase family 64 domain	46839.59	8.76	54.9	13.04	--
PHATRDRAFT_49038	773		predicted permeases	312691.3	7.39	55	4.36	----
PHATRDRAFT_49815	718		predicted protein	17466.51	4.39	50.1		---
PHATRDRAFT_50705	787	FCPC	fucoxanthin-chlorophyll a-c binding protein C, chloroplastic	21305.99	4.63	137	31.2	---
PHATRDRAFT_54065	40	LHCB1	fucoxanthin chlorophyll a/c protein	21277.98	5.23	101	19.70	-
PHATRDRAFT_54086	456	atpD_2	predicted F0F1 ATP synthase subunit beta	53642.67	4.7	310	28.74	-
PHATRDRAFT_55192	640	ECHS1	hydratase enyol-CoA hydratase	28912.5	5.6	75	12.26	---
PHATRDRAFT_56626	125	ADE2	putative phosphoribosylaminoimidazole carboxylase	64153.74	6.03	77.2	14.47	---
PSAC_PHATC	899	psaC	photosystem I iron-sulfur center	9289.3	5.75	174	55.56	-

Locus tag, identification of predicted protein in NCBI, uinprot, and swiss-prot; Spot ID, protein spot numbered in Fig. 1; Annotation, protein description annotated by MASCOT ( http://www.matrixscience.com); Protein mass, molecular mass of predicted protein; pI, isoelectric point; Protein score, total ion score; Coverage, peptide sequence coverage percentage (%). Changes of protein expression levels are indicated with the following symbols representing fold changes (FC): ++++/---- > 5; 5 > +++/--- > 4; 4 > ++ /-->3; 3 > + /-> 2.

**Table 2:** Validation of differentially expressed proteins by qPCR.

**Protein**	**Primer**		**qPCR**	**2-DE**
**Locus tag**	**Forward (5’-3’)**	**Reverse (5’-3’)**	**FC**	**FC**
45757	GCCATTTATGCCGACACCT	TCCGATAGGCAGGCACATT	9.16	++++
12583	ACAAGGCTACCGAAGGCAA	GGCTTGGTGTTCTTGGCTT	−1.42	++++
28882	CATTTCGCCTATTGGTCGTAA	TCAACCCCAAGTCAGCAAAT	1.24	++++
45813	GTTTGACTCTGCCGATTGCT	CTGGCGTAAATAAAGGAGGTGT	1.02	+++
42406	AACAAGGCATTCCGTTTCG	CCAGGGTGTCAAAATAAGCG	−3.35	+
18893	TGTCTTCCATCCCATCAACC	CAAATCACGGGCGTACAAA	−1.88	- -
22680	AGGTCACTCTGCTGGTCATCA	TCGGCTTCAACACCGTTCT	−1.52	- - - -
20657	GTGAAGACTGCGAATGTGGG	CAGATTGGAGCGTTTCTGAGA	−1.16	- -
55192	TACCGCTACAAGAAGGACTACG	GCCGACATACCTTCCGTTT	−1.03	- - -
24238	GGTGAGCGTTTGACGATTGA	GGTCCATTGCCGTGTACTAGA	−1.89	- - - -

Each value corresponds to the mean of three biological sample performed in duplicates; β- actin was used as an internal control. FC, fold changes.
